# Atrial Fibrillation is Associated With Morphine Treatment in Female Breast Cancer Patients: A Retrospective Population-Based Time-Dependent Cohort Study: Erratum

**DOI:** 10.1097/01.md.0000484178.43400.c3

**Published:** 2016-05-13

In the articles “Atrial Fibrillation is Associated With Morphine Treatment in Female Breast Cancer Patients: A Retrospective Population-Based Time-Dependent Cohort Study”,^1^ Dr. Ming-Chia Lin's affiliation was not reported in full. Dr. Lin is affiliated with the E-Da Hospital. The articles have since been corrected online.

## References

[1] Lee CW-S Muo C-H Liang J-A Atrial fibrillation is associated with morphine treatment in female breast cancer patients: A retrospective population-based time-dependent cohort study. *Medicine*. 2016 95:e3102. 26986153 10.1097/MD.0000000000003102 PMC4839934

